# Longitudinal 10-year changes in dietary intake and associations with cardio-metabolic risk factors in the Northern Sweden Health and Disease Study

**DOI:** 10.1186/s12937-017-0241-x

**Published:** 2017-03-28

**Authors:** Anna Winkvist, Sofia Klingberg, Lena Maria Nilsson, Maria Wennberg, Frida Renström, Göran Hallmans, Kurt Boman, Ingegerd Johansson

**Affiliations:** 1 Department of Public Health and Clinical Medicine, Nutritional Research, Umeå University, Umeå, Sweden; 2 Department of Internal Medicine and Clinical Nutrition, Sahlgenska Academy, University of Gothenburg, Box 459, SE-405 30 Gothenburg, Sweden; 3 Section for Epidemiology and Social Medicine (EPSO), Department of Public Health and Community Medicine, University of Gothenburg, Gothenburg, Sweden; 4 Arcum, Arctic Research Centre at Umeå University, Umeå, Sweden; 5 Department of Biobank Research, Umeå University, Umeå, Sweden; 6 Department of Clinical Sciences, Genetic & Molecular Epidemiology Unit, Lund University, Malmö, Sweden; 7 Research Unit, Medicine-geriatric clinic Skellefteå, Department of Public Health and Clinical Medicine, Umeå University, Umeå, Sweden

**Keywords:** Diet intake, Healthy Diet Score, Dietary Inflammatory Index, Body mass index, Serum lipids, Sweden, Population-based, Northern Sweden Diet Database

## Abstract

**Background:**

Dietary risks today constitute the largest proportion of disability-adjusted life years (DALYs) globally and in Sweden. An increasing number of people today consume highly processed foods high in saturated fat, refined sugar and salt and low in dietary fiber, vitamins and minerals. It is important that dietary trends over time are monitored to predict changes in disease risk.

**Methods:**

In total, 15,995 individuals with two visits 10 (±1) years apart in the population-based Västerbotten Intervention Programme 1996–2014 were included. Dietary intake was captured with a 64-item food frequency questionnaire. Percent changes in intake of dietary components, Healthy Diet Score and Dietary Inflammatory Index were calculated and related to body mass index (BMI), serum cholesterol and triglyceride levels and blood pressure at the second visit in multivariable regression analyses.

**Results:**

For both sexes, on group level, proportion of energy intake (E%) from carbohydrates and sucrose decreased (largest carbohydrate decrease among 40 year-olds) and E% protein and total fat as well as saturated and poly-unsaturated fatty acids (PUFA) increased (highest protein increase among 30 year-olds and highest fat increase among 60 year-olds) over the 10-year period. Also, E% trans-fatty acids decreased. On individual basis, for both sexes decreases in intake of cholesterol and trans-fatty acids were associated with lower BMI and serum cholesterol at second visit (all *P* < 0.05). For men, increases in intake of whole grain and Healthy Diet Score were associated with lower BMI and serum cholesterol at second visit (all *P* < 0.05). Also for men, decreases in intake of trans-fatty acids and increases in Healthy Diet Score were associated with lower systolic blood pressure at second visit (*P* = 0.002 and *P* < 0.000). For women, increases in intake of PUFA and Healthy Diet Score were associated with lower BMI at second visit (*P* = 0.01 and *P* < 0.05). Surprisingly, increases in intake of sucrose among women were associated with lower BMI at second visit (*P* = 0.02).

**Conclusions:**

In this large population-based sample, dietary changes over 10 years towards less carbohydrates and more protein and fat were noted. Individual changes towards the Nordic dietary recommendations were associated with healthier cardio-metabolic risk factor profile at second visit.

## Background

In September 2015, the Global Burden of Disease Project demonstrated that dietary risks (including high intake of red meat and sugar-sweetened beverages and low intake of fruits and vegetables, whole grains and poly-unsaturated fatty acids) contributed to the largest proportion of disability-adjusted life years (DALYs) globally for both women and men [1]. Thus, dietary risks are today more important for health than high blood pressure, high body mass index or smoking. In total, it is estimated that 11.3 million deaths occur annually because of dietary risks [1]. Also in Sweden, dietary risks contribute to the largest proportion of DALYs [2]. Unfortunately, trends in dietary intake in many parts of the world are towards what has been called “the Industrial Diet”, i.e. a diet characterized by highly processed foods with a high content of saturated fat, sugar and salt and a low content of dietary fiber, vitamins and minerals [3]. It is therefore important that longitudinal changes in dietary intake of populations are monitored, if we are to understand the association between dietary risks and health outcomes and to be able to reverse the worrisome trend.

Few population-based studies with detailed information on dietary intake and health exist and especially where diet intake has been monitored repeatedly in the same individuals. The Northern Sweden Diet Database (NSDD) is the largest such database, based on one population, in Europe (http://www.biobank.umu.se/biobank/biobank---for-researchers/northern-sweden-diet-database/), with detailed information on over 100 000 unique individuals. Approximately one third of them have participated a second time 10 years after the first visit, thus creating a unique cohort with repeated diet and health information. The aim of the current research was to report on longitudinal changes over a 10-year period in dietary intake for almost 16,000 individuals with complete data, and to evaluate associations between individual changes in dietary components of special health relevance and cardio-metabolic risk factors at the second study visit.

## Research design and methods

### Study population

The Västerbotten Intervention Programme (VIP) is an ongoing, population-based prospective study. Dietary intake data from participants of VIP makes up the major part of NSDD [4]. Since 1985, inhabitants of Västerbotten County in northern Sweden (total population about 255 000) have been invited by their local health center for a medical examination when turning 40, 50 and 60 years old. In some communities, 30 year-olds were also included until 1996 [5]. Over the years, between 48 and 67% of available inhabitants have participated in VIP [6]. Little evidence of selection bias has been found [7] and cancer incidences in the VIP cohort and in the general population of Västerbotten are basically identical, indicating a truly population based cohort [8].

### Diet measurements

At the visit, participants donate fasting venous blood samples and fill in an extensive diet and lifestyle questionnaire [9], including a semi-quantitative food frequency questionnaire (FFQ) that covers the preceding 12 month period [10]. Initially, the FFQ included 84 food items but from 1996 a short 64, 65 or 66 food item version was implemented. This reduction was achieved by deleting entire foods that were less common (e.g. liver and kidney) or in a few cases by merging similar food items. For the current analyses, only participants with the 64 item FFQ at both baseline and the follow-up were eligible.

In the FFQ, frequency of intake is reported on a 9-level scale from “never” to “≥4 times per day”. Daily intake in gram/day has been calculated by multiplying reported frequency of intake by a portion size value using the national food composition database [11]. Portion sizes for staple food, meat and vegetables are estimated from color photographs of four plates of increasing portion sizes. For other foods, sex- and age-specific average portion sizes as reported in a validation study in a subset of VIP participants using 24-h recall interviews [10] or as reported by the Swedish Food Agency [12] have been used. In addition to the validation study by 24-h recall interviews [10, 13], the FFQ also has been validated by serum biomarkers [14, 15].

A Healthy Diet Score that reflects healthy eating habits was calculated as previously described [16]. Briefly, frequency of intake per day was calculated for eight food/beverage groups. Favorable food groups included fish, fruits (except juices), vegetables (except potatoes) and whole grains. Unfavorable food/beverage groups included red or processed meats, desserts and sweets, sugar-sweetened beverages and fried potatoes. Intake frequencies were ranked within each sex in ascending quartile ranks for favorable foods/beverage groups, and in descending quartile ranks for unfavorable foods/beverage groups. The sum of all quartile ranks represents the Healthy Diet Score, with a minimum of zero and a maximum of 24 and with higher ranks indicating healthier food and beverage choices.

Further, a Dietary Inflammatory Index (DII) was calculated as suggested by Shivappa and colleagues [17], but modified to fit the population under study (Bodén S, personal communication). Here, 30 food parameters available in the data from the FFQ were used, out of the 45 food parameters suggested by Shivappa and colleagues [17]. First, individual intake of the 30 food parameters were expressed as z-scores relative to global intake data provided by Shivappa and colleagues. In this modified version of DII, contributions from coffee and caffeine in tea were combined into one caffeine parameter although tea was also kept as a food parameter of its own. Thereafter, estimated inflammatory contributions of each food parameter were calculated, based on literature-derived overall inflammatory effect-values also provided by Shivappa and colleagues. Food parameters known to contribute to inflammation yield positive values and food parameters known to prevent inflammation yield negative values. Finally, contributions from all consumed food parameters were summed to a final Dietary Inflammatory Index, with low values representing anti-inflammatory actions and high values representing pro-inflammatory actions.

### Lifestyle and health variables

Physical activity was measured using the Cambridge index of Physical Activity [18]. This is a validated index based on one question on occupational physical activity and one question on leisure time physical activity. Participants were categorized into inactive, moderately inactive, moderately active and active, respectively. Smoking was categorized into current smoker, ex-smoker, and never smoker, respectively. Serum cholesterol and triglycerides were measured at the health centers using a Reflotron bench top analyzer (Boerhinger Mannheim GmbH Diagnostica, Germany) until September 9, 2009. After that date, measurements were performed using an enzymatic routine method at the Department of Clinical Chemistry at the nearest hospital. Serum cholesterol values measured with Reflotron were calibrated to values corresponding to the enzymatic method using the algorithm: cholesterol_corrected_ = 0.738 + (0.901*cholesterol_Reflotron_). Similarly, serum triglyceride values measured with Reflotron were calibrated to values corresponding to the enzymatic method using the algorithm: triglycerides_corrected_ = 0.888 + (0.139*triglycerides_Reflotron_). Blood pressure was measured once, after 5 min rest and in supine position, using a sphygmomanometer.

### Sample selection

Between January 1990 and January 2014, in total 40,066 unique individuals had two visits recorded in VIP. Among these, all individuals who at both visits had filled in the short version of the food frequency questionnaire (64–66 items; see below) were selected (*n* = 17,461). Thereafter, 125 individuals were excluded because of too short or too long interval between the two visits; for these analyses intervals between visits were restricted to 10 ± 1 year only. The study sample thus included 17,336 individuals who had a first visit during 1996–2004 and a second visit during 2005–2014.

Among these 17,336 individuals, additional exclusions took place for the following reasons: individuals with >10% of the FFQ missing (*n* = 171 for first visit and *n* = 273 for second visit; *n* = 419 unique individuals excluded), individuals who had not filled in the three pictures indicating portion sizes (*n* = 145 for first visit and *n* = 168 for second visit; *n* = 263 unique individuals excluded) and individuals with food intake level (FIL; calculated by dividing reported total caloric intake with estimated basal metabolic rate [19]) below the 1st percentile or above the 99th percentile calculated separately by sex, or missing body weight so that FIL could not be calculated (*n* = 562 for first visit, *n* = 552 for second visit; *n* = 997 unique individuals excluded). Some individuals were affected by only one of these exclusion criteria and some individuals were affected by several of them. In total, *n* = 16,019 individuals had acceptable dietary information at both visits. Further, body weight (kg) and height (m) were measured in light clothing without shoes, by trained nurses using standardized weight and measuring scales. Body mass index (BMI) was calculated as weight/height^2^. Individuals with weight < 35 kg, length < 130 cm or BMI < 15 were excluded, rendering a final data set for analyses of *n* = 15,995.

### Data analysis

Mean values and standard deviations (SD) were calculated for intake of dietary components as well as Healthy Diet Score and Dietary Inflammatory Index for both sexes at both study visits, adjusted for age, BMI quintile within sex and year of examination. Intakes of dietary components were expressed as grams per day as well as percent of energy (E%). Intake of whole grain was expressed as gram per day as well as gram per 2000 kcal. To evaluate selection bias, socio-demographic characteristics and dietary intake data were compared between the study sample of *n* = 15,995 individuals and the entire VIP study population of *n* = 33,109 with at least one study visit during the same time period and applying the same exclusion criteria with respect to dietary intake data and anthropometry.

For the study sample, percent change over the 10 year period and 95% Confidence Interval (CI) of intake of dietary components, expressed as E%, were calculated by sex and age strata (30, 40, 50, or 60 year-olds at the time of the first visit), adjusted for sex-specific BMI quintile and year of examination.

Finally, multivariable regression analyses were performed with BMI, serum cholesterol, serum triglycerides or systolic blood pressure at the second study visit as outcome variable, respectively, representing important cardio-metabolic risk factors. Ten year percent changes in dietary components of special relevance to health (whole grain, poly-unsaturated fatty acids (PUFA), cholesterol, trans-fatty acids and sucrose) were evaluated as exposure variables. Correlations among these predictor variables were initially inspected to ensure that they could all be entered into the same model; all were < 0.3 and thus deemed acceptable except between cholesterol and trans-fatty acids (*r* = 0.6). Therefore, separate models were run with either cholesterol or trans-fatty acids as exposure variable. Finally, separate models for Healthy Diet Score as well as Dietary Inflammatory Index were evaluated, as these capture many aspects of the diet on their own. The regression models were all adjusted for values of the cardio-metabolic risk factor, year of study participation, age, education, smoking status and physical activity at the first visit. In sensitivity analyses, models were run with 10-year changes (as absolute values and as % change) in BMI, serum cholesterol, serum triglycerides or systolic blood pressure as outcome variable, respectively; these results were basically identical to the analyses described above and are not shown. For all regression models, R^2^ was calculated for the fully adjusted models as well as for models without non-dietary covariates to assess how much of the variation in outcome trait that is explained by the dietary variables. Level of significance was set to 0.05 and all analyses were carried out using IBM SPSS Statistics for Windows, Version 22.0. (Armonk, NY: IBM Corp.).

## Results

**Fig. 1:**
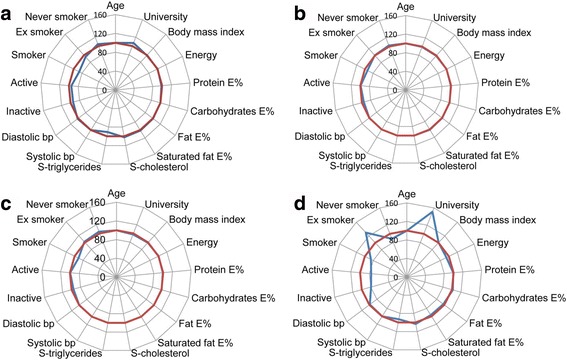
Star diagram illustrating phenotypic characteristics of the female study sample versus the female VIP population with at least one visit by age group. **a** 30-, **b** 40-, **c** 50- and **d** 60 year-old groups. *Red line* represents VIP population values set to 100% as reference and *blue line* represents study sample values

**Fig. 2:**
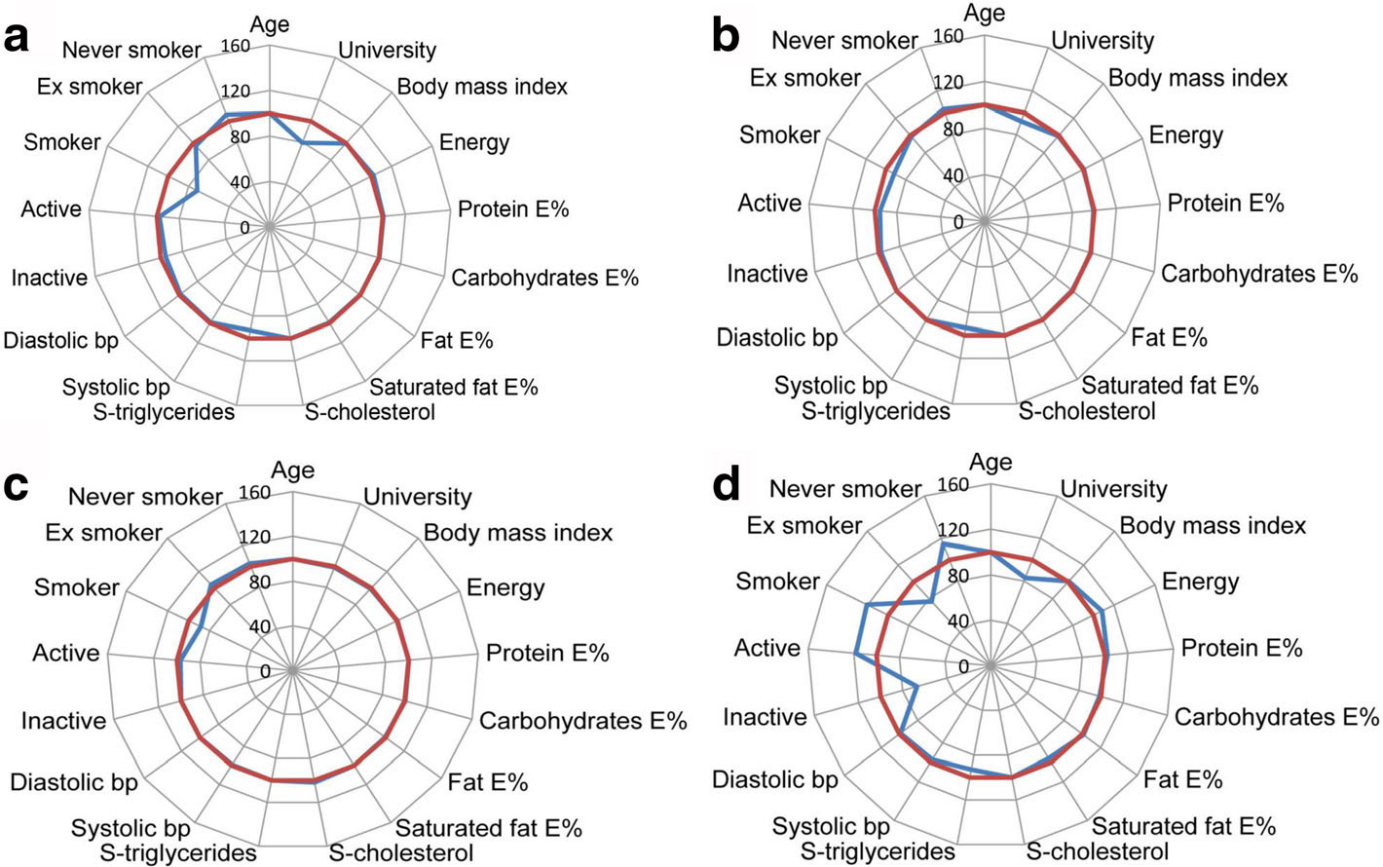
Star diagram illustrating phenotypic characteristics of the male study sample versus the male VIP population with at least one visit by age group. **a** 30-, **b** 40-, **c** 50- and **d** 60 year-old groups. *Red line* represents VIP population values set to 100% as reference and *blue line* represents study sample values

Overall, socio-demographic characteristics and dietary intake in the study sample of women (Fig. 1) and men (Fig. 2) with two repeat visits closely resembled the entire VIP population of women and men with at least one visit during the same time period. Exceptions were fewer female and male smokers in the study sample and that the very small study sample of 60-year-olds differed somewhat from the corresponding VIP population. Here, women in the study sample were more highly educated and more often ex-smokers, and men were more often smokers and more physically active.

For the study sample, during the 10 year period both women and men gained on average 2.5 kg and BMI increased one unit (Table 1). Serum cholesterol values were higher for women at the second visit, whereas this was not the case for men. For both sexes, serum triglyceride values and blood pressure were higher at the second visit. Both sexes reported being somewhat more physically active at the second visit and fewer individuals reported smoking.

**Table 1:** Socio-demographic and health characteristics among the 15,995 VIP participants with 10-year follow-up data^a^

Characteristic	Women (*n* = 8,354)		Men (*n* = 7,641)	
	At first visit	At second visit	At first visit	At second visit
Age (yrs)	44.6 ± 5.8	54.6 ± 5.8	44.6 ± 5.8	54.6 ± 5.8
Education (%)				
Secondary school or less	64.8	61.9	76.6	74.6
Academic education	35.2	38.1	23.4	25.4
Height (m)	165.6 ± 5.9	165.1 ± 6.0	179.1 ± 6.4	178.6 ± 6.5
Weight (kg)	68.8 ± 12.4	71.2 ± 13.0	83.8 ± 11.9	86.5 ± 13.2
Body mass index (kg/m^2^)	25.1 ± 4.3	26.1 ± 4.6	26.1 ± 3.3	27.1 ± 3.8
Serum cholesterol (mmol/l)	5.5 ± 0.9	5.8 ± 1.0	5.7 ± 1.0	5.7 ± 1.0
Serum triglycerides (mmol/l)	1.0 ± 0.1	1.1 ± 0.5	1.1 ± 0.1	1.3 ± 0.7
Systolic blood pressure (mmHg)	121.1 ± 15.9	126.0 ± 16.9	125.6 ± 14.2	130.4 ± 16.2
Diastolic blood pressure (mmHg)	75.4 ± 10.4	79.0 ± 9.9	79.0 ± 10.2	83.2 ± 1 + .1
Physical activity index (%)				
Inactive	17.2	15.8	18.6	16.9
Moderately inactive	34.3	27.6	32.9	28.5
Moderately active	27.8	26.6	28.7	29.1
Active	20.7	30.0	19.8	25.5
Smoking (%)				
Smoker	21.5	14.2	18.1	12.6
Ex-smoker	32.0	36.4	31.9	35.0
Never smoker	46.5	49.4	50.0	52.4

^a^Mean values (SD) adjusted for age and year of study participation (mean values for age only adjusted for year of study participation)

Reported energy intake was lower at the second visit for both women and men (Table 2). For both sexes, proportion of total energy intake from carbohydrates decreased, whereas proportion from protein and fat increased. For both sexes, proportion of energy intake from sucrose decreased, whereas reported intake of whole grain per 2000 kcal increased for women but decreased for men. Further, proportion of total energy intake from both saturated fat and PUFA increased for both sexes, whereas proportion from trans-fatty acids decreased. Proportions of saturated fat and PUFA from total fat intake hence remained unchanged over time for both sexes. No substantial change in DII over time was noted for either sex. As expected, no group-level change in Healthy Diet Score was found and this reflects that the score represents a mean of all ranks within the group at each time point.

**Table 2:** Intake of nutrients and diet indexes among the 15,995 VIP participants with 10-year follow-up data^a^

Item	Women (*n* = 8,354)		Men (*n* = 7,641)	
	At first visit	At second visit	At first visit	At second visit
Energy (kcal/day)	1546 ± 424	1368 ± 394	2033 ± 610	1854 ± 575
Protein (g/day)	55.7 ± 16.9	53.6 ± 17.3	70.0 ± 22.6	68.8 ± 23.4
Protein (E%)	15.0 ± 2.1	16.4 ± 2.5	14.5 ± 2.2	15.5 ± 2.5
Carbohydrates (g/day)	195.5 ± 59.8	161.6 ± 53.9	238.6 ± 81.0	206.6 ± 76.2
Carbohydrates (E%)	52.3 ± 6.1	49.0 ± 7.4	49.1 ± 6.6	46.1 ± 7.4
Whole grain (g/2000 kcal)	81.1 ± 34.7	88.0 ± 33.1	74.3 ± 30.3	71.5 ± 30.2
Sucrose (g/day)	24.4 ± 12.6	18.1 ± 9.4	32.2 ± 18.3	25.3 ± 16.0
Sucrose (E%)	6.5 ± 2.6	5.5 ± 2.2	6.6 ± 2.9	5.6 ± 2.8
Fat (g/day)	56.0 ± 18.6	52.2 ± 18.3	80.8 ± 29.0	78.2 ± 28.2
Fat (E%)	32.7 ± 5.9	34.6 ± 6.7	36.3 ± 6.5	38.3 ± 7.1
Saturated fat (g/day)	23.0 ± 8.5	21.4 ± 8.3	33.8 ± 13.4	32.8 ± 13.2
Saturated fat (E%)	16.4 ± 4.3	17.6 ± 5.3	19.1 ± 5.1	20.5 ± 6.0
PUFA^b^ (g/day)	9.0 ± 3.8	8.9 ± 3.9	12.9 ± 6.0	12.5 ± 6.1
PUFA (E%)	5.3 ± 1.6	5.9 ± 1.9	5.8 ± 2.0	6.1 ± 2.3
Trans-fatty acids (g/day)	1.5 ± 0.6	0.7 ± 0.4	2.3 ± 0.9	1.1 ± 0.5
Trans-fatty acids (E%)	0.9 ± 0.2	0.5 ± 0.2	1.0 ± 0.3	0.5 ± 0.2
Cholesterol (g/day)	0.15 ± 0.05	0.16 ± 0.06	0.22 ± 0.08	0.22 ± 0.09
Cholesterol (E%)	0.09 ± 0.02	0.1 ± 0.03	0.1 ± 0.02	0.1 ± 0.03
Healthy Diet Score	12.2 ± 4.0	12.0 ± 4.2	12.2 ± 3.8	11.8 ± 4.1
Diet inflammatory index	1.0 ± 1.7	1.0 ± 1.7	0.8 ± 1.8	0.9 ± 1.9

^a^Mean values (SD) adjusted for age, body mass index quintile within each sex and year of study participation

^b^ *PUFA* poly-unsaturated fatty acids

The four age groups (i.e., being 30, 40, 50 or 60 years old at first visit) exhibited similar patterns in percent changes in macronutrients over the 10-year period (Table 3). However, the youngest age group exhibited the largest percent decrease in reported energy intake and the largest percent increase in reported proportion of total energy intake from protein. The oldest age group exhibited the largest percent increase in reported proportion of total energy intake from fat and saturated fat. Women and men who were 40 years old at first visit had the largest percent decrease in reported proportion of total energy intake from carbohydrates.

**Table 3:** Percent change in intake of nutrients over the 10 year period for VIP participants^a^

	Percentage change (%)			
	30-year old	40-year old	50-year old	60-year old
Women (*n* = 8,354)	(*n* = 318)	(*n* = 3,937)	(*n* = 4,068)	(*n* = 31)
Energy (from kcal/day)	−12.0 (−14.8, −9,3)	−6.5 (−7.2, −5.7)	−10.8 (−11.5, −10.0)	+1.0 (−10.0, 12.1)
Protein (from E%)	+18.4 (16.1, 20.7)	+9.6 (9.0, 10.2)	+10.7 (10.1, 11.3)	+5.3 (−1.1, 11.8)
Carbohydrates (from E%)	−0.7 (−2.3, 0.9)	−7.5 (−8.0, −7.0)	−3.9 (−4.4, −3.5)	−5.9 (−9.8, −2.1)
Fat (from E%)	−2.2 (−4.5, 0.1)	+11.5 (10.7, 12.3)	+6.6 (5.8, 7.4)	+12.3 (4.8, 19.7)
Saturated fat (from E%)	+0.4 (−4.7, 3.9)	+16.3 (15.0, 17.6)	+11.5 (10.1, 13.0)	+21.1 (10.2, 32.0)
Men (*n* = 7,641)	(*n* = 340)	(*n* = 3,471)	(*n* = 3,808)	(*n* = 22)
Energy (from kcal/day)	−14.2 (−17.1, −11.2)	−7.4 (−8.3, −6.5)	−2.1 (−3.1, −1.2)	+0.4 (−11.8, 12.5)
Protein (from E%)	+10.7 (7.8, 13.7)	+7.4 (6.7, 8.0)	+9.3 (8.7, 7.7)	+1.4 (−7.6, 10.4)
Carbohydrates (from E%)	−3.0 (−4.8, −1.2)	−6.2 (−6.8, −5.7)	−4.1 (−4.6, −3.6)	−4.5 (−12.7, −3.8)
Fat (from E%)	+3.9 (1.8, 6.1)	+9.3 (8.6, 10.1)	+6.7 (5.9, 7.4)	+9.5 (−1.3, 20.4)
Saturated fat (from E%)	+3.8 (0.2, 7.4)	+12.9 (11.6, 14.1)	+12.5 (11.3, 13.8)	+19.2 (3.3, 35.2)

^a^Mean values (95% CI) adjusted for body mass index quintile within each sex and year of study participation

Significant associations between 10-year percent changes in dietary intake and cardio-metabolic risk factors at second visit were found (Tables 4, 5, 6 and 7). Decreases in dietary intake of cholesterol and trans-fatty acids were associated with significantly lower values for BMI and serum cholesterol at second visit for both sexes and for trans-fatty acids with lower systolic blood pressure for men (positive beta-values; all *P* < 0.05). Among women, increased intake of PUFA was significantly associated with lower BMI (*P* = 0.01). Increases in consumption of whole grain were associated with significantly lower BMI and serum cholesterol and a trend for triglyceride values at second visit for men (*P* < 0.000, *P* = 0.004 and *P* = 0.05). Increases in intakes of sucrose had no significant association with any of the cardio-metabolic risk factors at second visit, except with significantly lower BMI for women (*p* = 0.02).

**Table 4:** Change in body mass index (kg/m^2^) per percent diet intake increase over a 10-year period based on multivariable regression analysis^a,b^

	Women (*n* = 8,354)		Men (*n* = 7,641)	
	Beta ± SE	*P*	Beta ± SE	*P*
Joint model				
Whole grain	−0.02 ± 0.03	0.55	−0.13 ± 0.03	0.000
PUFA^c^	−0.18 ± 0.07	0.01	−0.07 ± 0.05	0.22
Cholesterol^d^	0.39 ± 0.07	0.000	0.14 ± 0.07	0.03
Trans-fatty acids^d^	0.55 ± 0.10	0.000	0.29 ± 0.11	0.008
Sucrose	−0.16 ± 0.07	0.02	−0.06 ± 0.04	0.18
Joint model R^2^	68.7%		72.0%	
Joint model R^2^ excluding covariates	1.4%		0.6%	
Separate models				
Healthy Diet Score	−0.42 ± 0.06	0.000	−0.31 ± 0.05	0.000
Joint model R^2^	68.7%		72.0%	
Joint model R^2^ excluding covariates	0.2%		0.0%	
Diet Inflammatory Index	0.000 ± 0.001	0.58	0.000 ± 0.001	0.44
Joint model R^2^	68.5%		71.9%	
Joint model R^2^ excluding covariates	0.0%		0.0%	

^a^Adjusted for body mass index, year of study participation, age, education, smoking status and physical activity at the beginning of the period

^b^All dietary items expressed as percent change over the 10 year period; sucrose, PUFA, cholesterol and trans-fatty acids calculated as energy percent, and whole grain calculated as gram per 2000 kcal

^c^ *PUFA* poly-unsaturated fatty acids

^d^Joint model run with either cholesterol or trans-fatty acids separately, because of high collinearity between the two. Results for other items in the model are shown for model run with cholesterol; values were basically identical for model run with trans-fatty acids

**Table 5:** Change in serum cholesterol (mmol/l) per percent diet intake increase over a 10-year period based on multivariable regression analysis ^a,b^

	Women (*n* = 8,354)		Men (*n* = 7,641)	
	Beta ± SE	*P*	Beta ± SE	*P*
Joint model				
Whole grain	−0.007 ± 0.01	0.52	−0.04 ± 0.02	0.004
PUFA^c^	−0.02 ± 0.02	0.35	−0.04 ± 0.02	0.11
Cholesterol^d^	0.16 ± 0.02	0.000	0.12 ± 0.03	0.000
Trans-fatty acids^d^	0.28 ± 0.03	0.000	0.20 ± 0.05	0.000
Sucrose	0.02 ± 0.02	0.43	0.001 ± 0.02	0.96
Joint model R^2^	27.8%		21.6%	
Joint model R^2^ excluding covariates	1.1%		0.1%	
Separate models				
Healthy Diet Score	−0.03 ± 0.02	0.10	−0.13 ± 0.02	0.000
Joint model R^2^	27.0%		21.4%	
Joint model R^2^ excluding covariates	0.00%		0.2%	
Diet Inflammatory Index	0.000 ± 0.000	0.60	0.000 ± 0.000	0.05
Joint model R^2^	26.9%		21.2%	
Joint model R^2^ excluding covariates	0.00%		0.00%	

^a^Adjusted for serum cholesterol, year of study participation, age, education, smoking status and physical activity at the beginning of the period

^b^All dietary items expressed as percent change over the 10 year period; sucrose, PUFA, cholesterol and trans-fatty acids calculated as energy percent, and whole grain calculated as gram per 2000 kcal

^c^ *PUFA* poly-unsaturated fatty acids

^d^Full model run with either cholesterol or trans-fatty acids separately, because of high collinearity between the two. Results for other items in the model are shown for model run with cholesterol; values were basically identical for model run with trans-fatty acids

**Table 6:** Change in serum triglycerides (mmol/l) per percent diet intake increase over a 10-year period based on multivariable regression analysis ^a,b^

	Women (*n* = 8,354)		Men (*n* = 7,641)	
	Beta ± SE	*P*	Beta ± SE	*P*
Joint model				
Whole grain	0.01 ± 0.006	0.09	−0.02 ± 0.01	0.05
PUFA^c^	−0.02 ± 0.01	0.50	−0.02 ± 0.02	0.32
Cholesterol^d^	0.01 ± 0.01	0.34	−0.01 ± 0.02	0.64
Trans-fatty acids^d^	0.02 ± 0.02	0.30	0.02 ± 0.04	0.62
Sucrose	−0.019 ± 0.01	0.13	−0.008 ± 0.02	0.60
Joint model R^2^	11.8%		11.5%	
Joint model R^2^ excluding covariates	0.2%		0.2%	
Separate models				
Healthy Diet Score	−0.03 ± 0.01	0.01	−0.05 ± 0.02	0.005
Joint model R^2^	11.6%		11.3%	
Joint model R^2^ excluding covariates	0.1%		0.00%	
Diet Inflammatory Index	0.000 ± 0.000	0.87	0.000 ± 0.000	0.99
Joint model R^2^	11.5%		11.2%	
Joint model R^2^ excluding covariates	0.00%		0.00%	

^a^Adjusted for serum triglycerides, year of study participation, age, education, smoking status and physical activity at the beginning of the period

^b^All dietary items expressed as percent change over the 10 year period; sucrose, PUFA, cholesterol and trans-fatty acids calculated as energy percent, and whole grain calculated as gram per 2000 kcal

^c^ *PUFA* poly-unsaturated fatty acids

^d^Full model run with either cholesterol or trans-fatty acids separately, because of high collinearity between the two. Results for other items in the model are shown for model run with cholesterol; values were basically identical for model run with trans-fatty acids

**Table 7:** Change in systolic blood pressure (mmHg) per percent diet intake increase over a 10-year period based on multivariable regression analysis ^a,b^

	Women (*n* = 8,354)		Men (*n* = 7,641)	
	Beta ± SE	*P*	Beta ± SE	*P*
Joint model				
Whole grain	−0.08 ± 0.18	0.68	−0.31 ± 0.23	0.19
PUFA^c^	−0.32 ± 0.40	0.42	0.005 ± 0.39	0.99
Cholesterol^d^	0.11 ± 0.39	0.78	0.94 ± 0.49	0.06
Trans-fatty acids^d^	0.60 ± 0.58	0.30	2.40 ± 0.79	0.002
Sucrose	−0.66 ± 0.38	0.08	0.38 ± 0.32	0.22
Joint model R^2^	20.3%		20.3%	
Joint model R^2^ excluding covariates	0.8%		0.2%	
Separate models				
Healthy Diet Score	−0.60 ± 0.32	0.06	−1.70 ± 0.39	0.000
Joint model R^2^	26.2%		20.4%	
Joint model R^2^ excluding covariates	0.00%		0.1%	
Diet Inflammatory Index	−0.006 ± 0.003	0.05	−0.001 ± 0.004	0.76
Joint model R^2^	26.2%		20.2%	
Joint model R^2^ excluding covariates	0.00%		0.00%	

^a^Adjusted for systolic blood pressure, year of study participation, age, education, smoking status and physical activity at the beginning of the period

^b^All dietary items expressed as percent change over the 10 year period; sucrose, PUFA, cholesterol and trans-fatty acids calculated as energy percent, and whole grain calculated as gram per 2000 kcal

^c^ *PUFA* poly-unsaturated fatty acids

^d^Full model run with either cholesterol or trans-fatty acids separately, because of high collinearity between the two. Results for other items in the model are shown for model run with cholesterol; values were basically identical for model run with trans-fatty acids

Increases in Healthy Diet Scores were associated with significantly lower values for BMI, serum cholesterol and triglyceride at second visit for both sexes (except for serum cholesterol for women where *P* = 0.10) and with lower systolic blood pressure for men (all *P* < 0.005). Finally, improvements in DII were not significantly associated with any of the cardio-metabolic risk factors at second visit.

In the regression models of BMI, serum cholesterol and triglyceride levels and systolic blood pressure at the second study visit, respectively, 10-year percent changes in dietary exposures as captured by sucrose, PUFA, cholesterol, trans-fatty acids and whole grain only explained 0.1–1.4% of the variation in the outcome trait (Tables 4, 5, 6 and 7). Likewise, percent changes in either Healthy Diet Score or Diet Inflammatory Index explained < 1.0% of the variance.

We performed two sensitivity analyses to address the potential confounding or mediating effect of BMI on the outcome trait. Firstly, we adjusted the regression models for baseline BMI but this did neither affect beta-estimates nor *p*-values (data not shown). Secondly, we adjusted the models for 10-year changes in BMI. For women, this attenuated the effect of Healthy Diet Score to non-significance for the outcomes serum cholesterol (*p* = 0.24), triglycerides (*p* = 0.074) and systolic blood pressure (*p* = 0.35). For men, the effect of whole grain on BMI was attenuated (*p* = 0.08) as well as the effect of dietary cholesterol on systolic blood pressure (*p* = 0.055).

## Discussion

In this large population-based cohort, longitudinal individual 10-year changes in intake of important health related dietary components during 1996–2014 were assessed. For both women and men, increased proportions of total energy intake from protein, total fat, saturated fat and poly-unsaturated fatty acids were demonstrated, concurrent with decreased proportions of total energy intake from carbohydrates, trans-fatty acids and sucrose. No substantial time trend in DII was noted. Importantly, for both sexes individual 10-year changes in dietary intake towards less cholesterol and trans-fatty acids and a better overall Healthy Diet Score were significantly associated with a more healthy cardio-metabolic risk profile at the second visit. Among men, increased consumption of whole grains and among women, increased consumption of PUFA also were beneficial for these outcomes. Changes in intake of sucrose and Diet Inflammatory Index had less pronounced effects on the outcomes studied.

### Methodological concerns and limitations

Evaluation of VIP participation has concluded that there is little evidence of selection bias and that the cohort is truly population-based [7]. The current study focuses on the sub-sample with two study visits available, 10 years apart. Comparison of this sub-sample with all VIP participants with at least one study visit during the same time period did not reveal any substantial selection bias, except that the very small sub-sample of 60 year-olds at first visit differed somewhat from their age-matched counterparts in the general VIP population. Hence, our study sample may be regarded as population-based, except for the oldest age group.

All age groups of both sexes, except the small group of 60 year at baseline, reported a lower energy intake at the second visit. This is likely due to a more pronounced under-reporting, in light of the increased BMI together with increased level of reported physical activity at the second visit. A possible reason for this finding may be the trend of larger portion sizes over time in Sweden as well as other rich societies [20]. Portion size trends are not captured in the diet intake assessment methodology used in the present study, where portion sizes for foods other than staple food, meat and vegetable either were calculated from the validation study performed in 2002 or derived from national standard portion sizes. Another reason for increased under-reporting over time may be that newer food items are not captured by the FFQ. Due to the change in reported energy intake over time, changes in diet components over time are in the presented analyses expressed as energy percent or per 2000 kcal.

Statistical analyses of nutrition intake are faced with the challenge of collinearity among many variables. In the current analyses, intake of cholesterol and trans-fatty acid exhibited strong correlation. Hence, separate models were run for these dietary exposure variables. However, remaining nutrient variables in the models may exhibit some collinearity that may mask true associations of the exposures on the health outcomes investigated. At the same time, other lifestyle factors such as physical activity and smoking are commonly associated with diet quality. While adjusting for these potential confounders, the true effects of the diet exposures might have been further masked. We demonstrated several significant associations between diet exposures and cardio-metabolic risk factors and, if anything, these associations would thus be underestimated. Also, diet intake data were collected with the use of an FFQ that should reflect diet intake during the past 12 months. As with all retrospective data collection, recall bias is possible in that individuals affected by ill health may report their previous exposure differently hence attenuating any true association between diet and health.

Further, in the present analyses, we have not adjusted for use of cholesterol lowering medications. It is possible that participants who were informed of high serum cholesterol values at first study visit started medications thereafter, leading to spurious associations between change in serum cholesterol values and any change in diet. However, in our previous publication on cross-sectional trends in diet intake and health in this population, we demonstrated that adjustments for use of cholesterol lowering medications had virtually no effect on estimated levels of serum cholesterol [4]. Still, the possibility of this kind of bias in the current longitudinal analyses must be kept in mind.

Strengths of the current study include that it represents a large population-based cohort with little evidence of self-selection, where exposure and outcome data have been collected with thoroughly validated methodology that has been consistent over the two time-points of measurement, 10 years apart, for all individuals.

### Results in relation to published studies

The present study of longitudinal changes in dietary intake prospectively confirms most cross-sectional dietary intake trends that we have previously reported for the period 1986–2010 in the same population [4]. Also in the cross-sectional data, increased proportion of total energy intake from total fat and saturated fat concurrent with decreased proportion of total energy intake from carbohydrates were demonstrated for both sexes. Similar results of decreasing intake of carbohydrates and increasing intakes of fat over time were recently reported from the German National Nutrition Survey [21]. Most other European studies reporting on time-trends of consumption rely on repeated cross-sectional surveys, in line with our previous report [4]. Recently, results were published from a Belgian longitudinal study of 197 women and 373 men with repeated measurements 10 years apart on dietary intake, expressed as three dietary indices, and anthropometry and blood lipid levels [22]. Here, a significant association between increases in dietary indices and anthropometric parameters was found in men only, whereas no significant associations were found with blood lipids. Perhaps the sample size was too small to detect significant associations among the women; also, changes in the diet indices over time may have been too small.

Of interest to note were the differences in reported diet changes by age group. For both sexes, 30 year-olds at first visit reported the largest increase among the age groups in proportion of energy coming from protein; those 40 years old at first visit reported the largest decrease in proportion of energy coming from carbohydrates and those 60 years at first visit reported the largest increase in proportion of energy coming from total fat and saturated fat. Perhaps this reflects different sub-cultures and different lifestyles among different age groups in Sweden. More research is needed on factors affecting the different patterns of different age groups.

Surprisingly, no changes over time were visible in the DII for either sex in the northern Swedish population. Also in the German National Nutrition Survey, no changes in Healthy Eating Index were visible over time for either sex [21]. The German researchers speculate that this may reflect simultaneous changes in consumption of healthy and less healthy food groups that may cancel each other out, making such index unsuitable for evaluating longitudinal trends in diet quality. The same likely holds for our results. Also, the DII does not account for level of energy intake. In our data, underreporting of energy intake likely increased at second examination. This may have attenuated an impact of individual increases in intake of food items with pro-inflammatory effects over time. Healthy Diet Score was calculated based on ranks within the group at each time point, meaning that group-level changes over time could not be detected. Importantly, individual improvements in Healthy Diet Score were associated with improved cardio-metabolic profile.

In a previous smaller longitudinal study of weight development among women only in the same VIP population, we have shown that high intake of fruit per se was inversely associated with incident obesity over 10 years [23]. In the present study we did not investigate change in fruit intake as a single predictor of BMI. Still, fruit is part of the Healthy Diet Score and, in line with the previous finding [23], we here demonstrate an increased score to be associated with lower BMI at the second visit for both women and men. Other studies relating change in diet exposure to weight outcome yielded results in accordance with ours. Field et al. [24] showed in the Nurses’ Health Study in the US that increases in percentage energy from saturated fat and trans fat were positively associated with weight gain over 8 years and Newby et al. [25] showed in a Swedish female cohort that increase in a healthy diet pattern score was inversely associated with weight gain over 9 years. In both studies, the effects were stronger in women with higher baseline BMI. On the contrary, Togo et al. [26] did not find any significant association between change in food intake pattern and change in BMI or obesity development in Danish women and men. However, that study may not have fully captured the dietary exposure in that the FFQ was limited to 26 items only. Taken together, the findings from our study and those cited above indicate that a healthy diet supports healthy weight development.

The present longitudinal study confirms the recent trends of increases in dietary total and saturated fat intake concurrently with increases in serum cholesterol levels (for women) and BMI (for both sexes), which we noted in cross-sectional data from the same population [4]. The significant associations between decreased dietary intake of cholesterol and trans-fatty acids as well as increased Healthy Diet Score and lower serum cholesterol levels and lower BMI warrant attention. Based on our results, a decreased consumption of cholesterol with 10% would be associated with a decrease in serum cholesterol levels with 1.6 mmol/l for women and 1.2 mmol/l for men, and with 3.9 lower BMI units for women and 1.4 lower BMI units for men.

Still, the overall explanatory power of the dietary exposures studied was small and the associations reported must be interpreted with some caution. However, as discussed above the associations are most likely attenuated by random errors and collinearity in the diet exposure variables and therefore in reality somewhat larger. On a population level, dietary changes towards higher accordance with current dietary guidelines would likely have a substantial positive effect on public health. For example, Valsta and co-workers [27] estimated that changes in dietary fat quality and cholesterol intake might explain as much as 65% of the decrease in serum cholesterol levels seen in Finland between 1982 and 2007, illustrating the potential of dietary changes in affecting cardio-metabolic risk factors on a population-level. Further, Björck et al. [28] used the IMPACT model to simulate effects of changes in dietary fat intake on predicted total blood cholesterol levels and coronary heart disease mortality in Sweden between 2010 and 2025. Population-based values for predicted changes in the risk factors smoking, salt intake and physical inactivity were included in the models. Thereafter, two different levels of saturated fat intake were modelled: 10 energy percent representing current Nordic Nutrition Recommendations, or 20 energy percent representing current trends in Sweden of increased intake of saturated fat. The latter scenario predicted in total 400 additional deaths from coronary heart disease in 2025 than did the former scenario.

Further, it is possible that the effects of diet exposure on serum cholesterol, triglycerides and blood pressure were indeed confounded or mediated through effects on BMI. Sensitivity analyses showed that the effects were not confounded by baseline BMI, but that change in BMI seemed to, at least partially, mediate the effect of Healthy Diet Score on serum cholesterol, triglycerides and systolic blood pressure in women.

Few associations were found between reported changes in intake of sucrose and cardio-metabolic risk factors. This is surprising in that high intake of sucrose in randomized controlled trials has been associated with increased levels of serum cholesterol and triglycerides as well as higher blood pressure, independent of effects on weight [29]. Similar results also have been found in observational studies [30]. Our result of an association between increased consumption of sugar and lower BMI among women may be a chance finding, since it is not supported by corresponding findings in the literature or by biological explanations. This warrants further investigations.

## Conclusion

In this large population-based cohort study, group level dietary changes over 10 years towards less carbohydrate and more protein and fat intakes were noted as well as a decrease in trans fatty acids and sucrose consumption. For the individual, dietary changes in line with current Nordic dietary recommendations were associated with a more favorable cardio-metabolic profile and lower BMI at the second visit, independent of baseline values of the studied outcomes.

## Abbreviations

BMI: Body mass index (kg/m2)
BMR: Basal metabolic rate
CI: Confidence interval
DALYs: Disability-adjusted life years
DII: Diet Inflammatory Index
E%: Energy percentage
FFQ: Food frequency questionnaire
FIL: Food intake level (calculated by dividing reported total caloric intake with estimated basal metabolic rate)
NSDD: The Northern Sweden Diet Database
PAL: Physical activity level (calculated by dividing estimated total energy expenditure with BMR)
PUFA: Poly-unsaturated fatty acids
SD: Standard deviation
VIP: Västerbotten Intervention Programme
